# Application of Nanoparticles for Oil Recovery

**DOI:** 10.3390/nano11051063

**Published:** 2021-04-21

**Authors:** Ole Torsæter

**Affiliations:** Department of Geoscience and Petroleum, Norwegian University of Science and Technology (NTNU), 7031 Trondheim, Norway; ole.torsater@ntnu.no

Due to their large surface-area-to-volume ratio and enhanced chemical reactivity, nanoparticles have attracted interest among researchers in the upstream petroleum industry for oil recovery applications. Nanoparticles have been studied as additives to waterflooding from day one of production as well as additives at later-stage waterflooding (secondary and tertiary recoveries). Many types of nanoparticles have been tested, and the aims of the nanoparticles have been either to reduce the mobility of the injected fluid relative to that of oil or to increase the ratio of viscous to interfacial forces (i.e., capillary number).

The research on nanotechnology for oil recovery has shown potential, but the mechanisms for oil recovery are not fully understood. This Special Issue of *Nanomaterials*, “Application of Nanoparticles for Oil Recovery”, includes eight research articles that demonstrate advances in the understanding of the behavior of nanoparticles during fluid flow in porous media and give more knowledge of recovery mechanisms when using nanoparticles in oil recovery. The research works presented here cover core flooding and microfluidic studies of the injection of various nanoparticle suspensions (in water and together with other chemicals) for enhanced oil recovery (EOR), nanostabilized CO_2_ foam flooding, investigation of stability of nanosuspensions under various conditions, and mathematical modelling of injection processes of polymer with nanoparticles.

Studies on the use of surface-functionalized particles for special reservoir rock and fluid properties and reservoir conditions might be the way forward for understanding the oil recovery mechanisms involved. An article in this Special Issue by Bila and Torsæter [1] investigated tertiary flooding of polymer-coated silica nanoparticles for oil recovery at high temperature and high salinity. The flooding tests indicated incremental oil recovery of up to 6% of the original oil in place (OOIP). Among the recovery mechanisms studied, the change in rock wettability to more water-wet seemed to be most important.

The stability of nanoparticles is important for a successful application of nanofluids for enhanced oil recovery. Hadia et al. [2] studied the stability of commercial silica nanoparticles in combination with zwitterionic and hydrophilic silanes under high salinity and high temperature conditions. The results showed thermal stability in synthetic seawater at 60 °C for 1 month and accelerated stability analysis predicted that the modified nanoparticles could remain stable for at least 6 months.The ability of the nanoparticles to alter rock wettability was also studied. The results showed that the modified nanoparticles were able to adsorb on rock surfaces and altered wettability to water-wet.

Previous research has shown that nanoparticles can increase the stability of surfactant-based foams. Stable foams are especially important in processes where CO_2_ foam is used to recover oil and in CO_2_ storage. The behavior of nanoparticle–surfactant foam formulations is not well understood, and the experimental work by Alcorn et al. [3] presents a pore-to-core-scale study of foam behavior. Snap-off was the most important foam generation mechanism in high-pressure micromodels. It was also observed that foaming solutions containing only nanoparticles generated very little foam. When nanoparticles and surfactant were used together, foam generation and strength were not sensitive to the nanoparticle concentration. The experiments with oil showed that the apparent viscosity of foam was nearly three times higher than that in the experiments without oil. This was due to the development of stable oil/water emulsions.

Davarpanah [4] presents a mathematical model for simultaneous injection of polymer-assisted nanoparticles for calculation of an oil recovery factor. A sensitivity analysis is provided to show the influence of formation damage in a nanoparticles–polymer solution injection process. The study shows that large mobility ratio, high polymer concentration, and more formation damage lead to increased recovery factor. The reason for this is that the external filter cake is building up in this period and the subsequent injection of the polymer solution thereby gives a better sweep efficiency and higher oil recovery.

The stability of a nanoparticle suspension at reservoir conditions is still creating uncertainty in the evaluation of nanoflooding processes. Li et al. [5] studied the effect of nanoparticle treatment on the stability at reservoir conditions in the presence of reservoir rock and crude oil. The stability was screened in test tubes at 70 °C and 3.8 wt. % NaCl. Fumed silica nanoparticles in suspension with hydrochloric acid (HCl), polymer-modified fumed nanoparticles, and amide-functionalized silica colloidal nanoparticles were studied. The results showed that both HCl and polymer surface modification can improve nanoparticle stability. It was also found that pH is important for nanoparticle stability. Based on the results in this study, a stabilizer and/or nanoparticles modification are necessary for EOR application.

The experimental study by Rueda et al. [6] showed the effect of adding nanoparticles in biopolymer flooding. The tests were performed in water-wet microfluidics setup using xanthan gum and scleroglucan, as well as silica-based nanoparticles in a secondary flooding mode where the capillary number was kept constant. The reference nanofluid flood resulted in higher ultimate oil recovery than similar floods with xanthan gum, scleroglucan, and brine. When adding nanoparticles to the biopolymer solutions, nano-xanthan flooding achieved the highest oil recovery. A reduced polymer adsorption in the nano-xanthan flooding may explain the improvement in the sweep efficiency and recovery factor.

Aadland et al. [7] conducted core flood and microfluidics experiments with fluids containing cellulose nanocrystals (CNCs) and 2,2,6,6-tetramethylpiperidine-1-oxyl (TEMPO)-oxidized cellulose nanofibrils (T-CNFs). Both particles were mixed in brine, and the oil recovery was compared with standard water flooding. CNCs produced 5.8% more of the original oil in place (OOIP) than pure brine flooding. The effect of injection scheme, temperature, and rock wettability was also investigated for both the CNC injection and brine injection, and CNC performed better than pure brine in all cases. The study of T-CNFs showed that these particles were even more effective than CNCs. However, the injectivity gradually deteriorated, and work is ongoing to solve this problem.

Rezaei et al. [8] investigated the effect of the pore throat size distribution on oil recovery by surfactant–nanofluid injection. Interfacial tensions and contact angles were measured to determine the optimum concentrations of an anionic surfactant and silica nanoparticles for core flooding experiments. The results of relative permeability tests showed that the pore throat size distribution affected the endpoints of the relative permeability curves, and a large amount of unswept oil was recovered by the flooding surfactant and silica nanoparticles. The results of the core flooding tests indicated that the injection in tertiary mode increased the post-water flooding oil recovery by up to 2.5% for carbonate core plugs with homogeneous pore throat size distribution and 8.6% for cores with heterogeneous pore throat size distribution.
